# Genome-wide identification of *CONSTANS*-LIKE genes and functional analysis of *FaCOL57* and *FaCOL59* in regulating anthocyanin and sugar synthesis in cultivated strawberry

**DOI:** 10.1186/s12864-026-12778-9

**Published:** 2026-03-26

**Authors:** Lixia Sheng, Xingming Zhu, Hao Zhu, ChangXu Duan, Jianqiang Yu, XiaoYing Zhu, Rui Lu

**Affiliations:** 1 College of Horticulture and Landscape Architecture, Yangzhou University, Yangzhou, 225009 China; 2 Zhongshan Biological Breeding Laboratory, Nanjing, Jiangsu 210000 China

**Keywords:** *Fragaria* × *ananassa*, *CONSTANS*-*like*, Anthocyanin, Soluble sugar, Temperature regulation, Floral transition

## Abstract

**Background:**

Cultivated strawberry (*Fragaria × ananassa*) is highly sensitive to temperature fluctuations, which influence floral induction, fruit ripening, and quality formation. CONSTANS-LIKE (COL) transcription factors are central regulators of photoperiod-dependent flowering and stress responses; however, their roles in temperature-mediated fruit quality regulation in octoploid strawberry remain unclear.

**Results:**

In this study, 63 *FaCOL* genes were identified in the octoploid strawberry genome and systematically characterized through phylogenetic analysis, gene structure, conserved motifs, cis-element composition, and synteny relationships. Transcriptome profiling of fruits exposed to low (6 °C) and high (26 °C) temperatures revealed distinct temperature-responsive expression patterns among *FaCOL* members. Notably, *FaCOL57* and *FaCOL59* exhibited antagonistic responses: *FaCOL57* was induced by cold but repressed by heat, whereas *FaCOL59* showed the opposite trend. Tissue-specific expression analysis indicated preferential accumulation of *FaCOL57* transcripts in floral tissues and *FaCOL59* in ripening fruits. Functional analyses demonstrated that virus-induced gene silencing of either gene significantly reduced anthocyanin and soluble sugar contents, while transient overexpression enhanced the accumulation of anthocyanin, sucrose, glucose, and fructose. Promoter analysis of key metabolic genes identified multiple canonical COL-binding elements, suggesting potential transcriptional regulation.

**Conclusions:**

These findings demonstrate that *FaCOL57* and *FaCOL59* function as positive regulators of anthocyanin and soluble sugar accumulation in cultivated strawberry and exhibit contrasting temperature-responsive expression patterns. Our results expand the functional diversity of the *FaCOL* gene family in octoploid strawberry and *FaCOL57* and *FaCOL59* may function as transcriptional nodes capable of integrating cold cues with metabolic outputs. This study establishes a foundation for further mechanistic dissection of *FaCOL*-mediated environmental signal integration and offers potential molecular targets for improving fruit quality stability under variable temperature conditions.

**Supplementary Information:**

The online version contains supplementary material available at 10.1186/s12864-026-12778-9.

## Introduction

The cultivated strawberry (*Fragaria* × *ananassa*) is the world’s most popular and economically important berry crop and is highly appreciated for its sweet aroma, juicy texture, nutrient content, and short lifecycle. Fruit sweetness and coloration are two key determinants of consumer preference and market competitiveness. Sucrose, the predominant transport carbohydrate, is translocated from source leaves to the receptacle. Upon cell entry, sucrose is either stored as vacuolar starch or hydrolyzed by invertase into glucose and fructose [1]. Accordingly, sucrose, glucose, and fructose constitute the major soluble sugars in ripe fruits. Genetic modulation of sucrose metabolicsm enzymes alters carbohydrate partitioning and accumulation [1]. For example, the manipulation of ADP-glucose pyrophosphorylase and sugar transporter *FveTST1* increased soluble sugar levels [2, 3].

Strawberries are also praised for their health-promoting anthocyanins, which confer fruit color and influence purchasing decisions. Anthocyanin biosynthesis proceeds from phenylalanine through sequential catalysis by *phenylalanine ammonia-lyase* (*PAL*), *chalcone synthase* (*CHS*), *chalcone isomerase* (*CHI*), *flavanone 3-hydroxylase* (*F3H*), *flavonoid 3′-hydroxylase* (*F3′H*), *flavonoid 3′*,*5′-hydroxylase* (*F3′5′H*), *dihydroflavonol 4-reductase* (*DFR*), *Anthocyanidin reductase* (*ANS*), and *UDP-glucose: flavonoid 3-O-glucosyltransferase* (*UF3GT*). Additionally, transcription factors FaMYB10, FaMYB1, and FaRIF orchestrate pathway flux by regulating structural gene expression [4–8].

Strawberry reproductive development and fruit maturation are strongly influenced by environmental cues, particularly temperature and photoperiod. Floral initiation marks the transition from vegetative to reproductive growth in strawberry, determining the onset of flowering and inflorescence number, and is thus a critical factor affecting market timing and yield. Based on flowering habits, strawberries are roughly categorized into two groups: seasonal flowering (SF) strawberries, induced by short days (SD) or cool temperatures, or perpetual flowering (PF) strawberries, which flower continuously under long days (LD) [9]. Most commercial genotypes are SF, with temperature as the dominant environmental cue, interacting with photoperiod. Both SF and PF types exhibit day-neutral flowering below 10–13 °C but suppression above 23–25 °C, even under inductive photoperiods [10–12]. In production, nursery transplants (September–November) encounter cool SD conditions that trigger floral induction. However, chilling during anthesis reduces pollen viability and impairs fertilization [13–15]. During fruit ripening, temperatures below 10 °C delay maturation, impair anthocyanin accumulation, and reduce soluble sugar content, ultimately leading to decreased fruit quality and market value [7, 16]. Thus, strawberries display stage-specific thermal optima. Despite the recognized importance of temperature responses in strawberry cultivation, the molecular mechanisms linking environmental temperature signals to reproductive development and fruit quality regulation remain poorly understood.

The protein CONSTANS (CO) from of Arabidopsis, which was the first one identified, constitutes the central regulator of the *CONSTANS-like* (*COL*) family in the photoperiod pathway. *CO* integrates circadian rhythms, light signaling, and development-related genes to regulate flowering time, playing a crucial role in floral induction [16–19]. The typical COL proteins contain two conserved domains: the N-terminal B-Box zinc finger domains (BBX) critical for protein–protein interactions and a C-terminal CCT domain involved with transcriptional regulation and nuclear localization [19]. COLs bind G-box (CACGTG/T), CCAAT, CORE1, and CORE2 motifs in target promoters [20–22]. Further, AtCO forms head-to-tail oligomeric structures via its B-box and phase-separates into CO/NF-YB2/NF-YC9/FT condensates to mediate *FT* activation under LD conditions [23, 24]. More than flowering, CO participates in diverse processes beyond flowering [25] including seed size [26], hormone responses [27], stomatal opening [26, 28], stress [27], lipid metabolism [29], carbon metabolism [30], and floral organ senescence [31]. Furthermore, as transcription factors, COL proteins are involved in plant morphogenesis and abiotic stress responses [32–38]. Examples include Arabidopsis *AtCOL3* positively regulates photomorphogenesis and promotes root growth, while rice *OsCOL9* modulates resistance to blast disease via salicylic acid and ethylene signaling [39]. *COL* genes in Arabidopsis, soybean, cannabis, foxtail millet, sand pear, longan, and mango play important roles in drought and salt stress responses [25, 27, 33, 40–42]. The diploid woodland strawberry (*F. vesca*) *COL* family comprises 10 genes, among which *FvCO* promotes flowering and inhibits stolon development [43, 44]. Temperature is a critical environmental factor affecting both floral transition and fruit quality formation in strawberry. However, the functional diversity and regulatory roles of *COL* genes in octoploid cultivated strawberry, particularly in temperature-mediated fruit quality formation, remain largely unexplored.

In this study, we performed genome-wide identification and characterization of the COL gene family in octoploid cultivated strawberry. We further identified two temperature-responsive genes, *FaCOL57* and *FaCOL59*, and investigated their positive regulation of anthocyanin biosynthesis and sugar accumulation using transcriptomic analysis, expression profiling, transient overexpression in strawberry fruits, and virus-induced gene silencing. Our findings provide new insights into the molecular mechanisms by which COL transcription factors integrate temperature signals to regulate reproductive development and fruit quality formation in strawberry.

## Materials and methods

### Plant materials

The strawberries used in the experiment were obtained from the greenhouse at the College of Horticulture and Landscape Architecture, Yangzhou University. They were cultivated in a substrate with nutrient solution irrigation, where the substrate ratio was peat: vermiculite: perlite = 2:1:1. For temperature treatment experiments:*Fragaria* x *ananassa* ‘Benihoppe’ at the white developmental stage (defined as fruits with fully expanded size but lacking visible red pigmentation) were harvested from commercial cultivation plots at Shatou Strawberry Picking Garden (Yangzhou, China). Fruits with uniform size and absence of mechanical damage or pathogen infection were selected for subsequent analyses.

### Identification of the *FaCOLs* in octoploid strawberry

To identify the members of the *FaCOL* gene family in the octoploid cultivated strawberry (2n =8x = 56), the whole-genome sequence and annotation files of cultivated strawberry ‘Benihoppe’ were downloaded from the Rosaceae Genome Database (GDR; https://www.rosaceae.org/*).* The hidden Markov model (HMM) profile for the COL protein domain (PF06203) was obtained from the Pfam database. TBtools software was used to identify *FaCOLs* in the octoploid strawberry genome [45], with an E-value threshold of ≤ 1 × 10⁻⁵. Redundant sequences identified by both HMM search and BLASTP were removed. Candidate proteins were further validated for the integrity of conserved domains using the SMART (https://smart.embl.de) and NCBI Conserved Domain Database (CDD; https://www.ncbi.nlm.nih.gov/Structure/cdd/*)* tools. Finally, all members of the *FaCOL* gene family were obtained.

### Structural, motif, and promoter analysis of *FaCOL* genes

TBtools software v2.154 [46] was used to analyze the gene structure information of the *FaCOLs* in cultivated strawberry, and the NCBI online tool was employed to analyze the *FaCOL* structures. The MEME (https://meme-suite.org/meme/tools/meme) website was utilized to identify conserved motifs in the proteins encoded by the *FaCOLs*, and TBtools was used to visualize the protein structures, conserved motifs, and gene structures. TBtools was used to extract the 2,000 bp sequences upstream of the transcription start sites of *FaCOL* genes. PlantCARE (https://bioinformatics.psb.ugent.be/webtools/plantcare/html) was employed to predict cis-elements in the promoter regions, and the prediction results were visualized using TBtools.

### Phylogenetic and collinearity analysis

MEGA7 software was used to perform multiple sequence alignment of the *COL* gene families from strawberry, apple and Arabidopsis. The phylogenetic tree was constructed using the Neighbor-Joining (NJ) method, with parameters set to Poisson correction, pairwise deletion, and bootstrap testing (1,000 replicates). The phylogenetic tree was beautified using the TVBOT web tool (https://chiplot.online/tvbot.html). Based on BLAST results and gene location information, TBtools with MCScanX (v2.119) was used to identify collinear gene pairs, and Circos was employed to visualize the data. The Gene Location Visualization function in TBtools was used to perform chromosomal localization.

### Temperature treatments and transcriptome sequencing

#### Temperature treatment design

The fruit stages are divided into the small green stage, the large green stage, the white stage, the turning stage, and the red stage [47]. Fruits were harvested from at least three independent plants. Each fruit was considered one biological replicate. Fruits were incubated in controlled growth chambers at: 6 °C (low temperature), 16 °C (control temperature), and 26 °C (high temperature). Growth chamber conditions were maintained at: Relative humidity: 80%; Photoperiod: 16 h light / 8 h dark; Light intensity: 150 µmol m⁻² s⁻¹. Water was replaced every 3 days. Fruits were sampled after 6 and 12 days of treatment, immediately frozen in liquid nitrogen, and stored at − 80 °C.

#### RNA extraction and library preparation

Total RNA was extracted using a commercial plant RNA extraction kit. RNA integrity was assessed using agarose gel electrophoresis and Agilent 2100 Bioanalyzer. mRNA libraries were prepared using poly(A) enrichment and fragmentation followed by cDNA synthesis. Libraries were sequenced using the MGI DNBSeq-T7 platform to generate paired-end reads (150 bp). Total RNA was isolated using a standard RNA extraction kit according to the manufacturer’s instructions. RNA integrity and concentration were assessed using agarose gel electrophoresis and Agilent 2100 Bioanalyzer. Only RNA samples with RIN ≥ 7.0 were used for library construction following the manufacturer’s standard protocol. Transcriptome sequencing was performed by Biomarker Technologies Corporation (Qingdao, China) using the MGI DNBSeq-T7 platform to generate paired-end reads (150 bp).

#### RNA-seq data processing

Raw sequencing reads were subjected to quality filtering using fastp to remove low-quality reads. The filtered clean reads were aligned to the reference genome of *Fragaria* × *ananassa* (GDR, https://www.rosaceae.org/) using HISAT2 with default parameters. Only uniquely mapped reads were retained for downstream analyses. Gene expression levels were quantified using feature Counts and normalized as FPKM (Fragments Per Kilobase of transcript per Million mapped reads) values. Differential expression analysis between low or high-temperature-treated samples and control samples was performed using the DESeq2 package in R. Differentially expressed genes were identified using thresholds of |log₂Fold Change| ≥ 0.585 with adjusted *p* value / FDR < 0.05. All identified DEGs were annotated by comparison with multiple public databases, including NR, Swiss-Prot, Gene Ontology (GO), and Kyoto Encyclopedia of Genes and Genomes (KEGG). GO functional enrichment analysis and KEGG pathway enrichment analysis were performed using hypergeometric tests, and pathways with FDR < 0.05 were considered significantly enriched. The Venn diagram was generated to screen for target genes.

### Cloning of *FaCOL57* and *FaCOL59* in cultivated strawberry

Total RNA from mature strawberry fruits was extracted using the Takara Total RNA Extraction Kit. The extracted total RNA served as the template for first-strand cDNA synthesis using the Takara Total reverse transcription kit (RR047A), and the product was stored at -20 °C. Based on the gene sequences from the transcriptome data and analysis of the open reading frames using the NCBI website, two pairs of specific primers were designed to clone the full-length cDNA. PCR products were purified by gel extraction, ligated into the pEASY-Blunt Zero Cloning Vector, and transformed into DH5α competent cells. The recombinant plasmids were sequenced by Sangon Biotech. BLAST searches were performed against the NCBI nucleotide and protein databases to confirm successful cloning of the corresponding homologous genes, followed by plasmid extraction. Primers are listed in Supplementary Data Set4.

### Construction of VIGS silencing vectors

To avoid conserved domains, gene fragments of approximately 300 bp were selected and cloned using the aforementioned plasmids as templates. The *p*TRV2 vector plasmid was digested with BamHI and SmaI restriction endonucleases, and the cloned fragments were inserted into the *p*TRV2 vector. The *p*TRV1 and *p*TRV2-*FaCOL* recombinant vectors were transformed into Agrobacterium tumefaciens GV3101. Single colonies containing *p*TRV1 and the recombinant *p*TRV2-*FaCOL* were selected and inoculated into 400 µL of LB liquid medium (containing 100 mg/L kanamycin and 50 mg/L rifampicin), then incubated at 28 °C with shaking at 200 rpm for 6 h. Subsequently, 200 µL of the bacterial suspension was added to 10 mL of LB liquid medium (containing 100 mg/L kanamycin and 50 mg/L rifampicin) and incubated at 28 °C with shaking at 200 rpm for 12 h. Bacterial cells were collected by centrifugation at 5,000 rpm for 6 min and resuspended in VIGS infiltration buffer (10 mmol/L MgCl₂, 10 mmol/L MES, 200 µmol/L acetosyringone). The cells were collected and resuspended in 100 mL of resuspension buffer (10 mM MgCl₂, 10 mM MES, 200 µM acetosyringone) to achieve an OD₆₀₀ of 2.0 for the recombinant vector infiltration solution. Primers are listed in Supplementary Data Set4.

### Agroinfiltration and silencing efficiency validation

‘Benihoppe’ strawberry fruits at the white stage were selected. A 1 mL syringe was inserted from the fruit tip into the hollow pith, and an equal-volume mixture of *p*TRV1 infiltration solution and *p*TRV2: *FaCOL* infiltration solution was slowly injected until the fruit surface appeared saturated. An equal-volume mixture of *p*TRV1 infiltration solution and empty *p*TRV2 infiltration solution served as the negative control (Mock). Strawberry fruits (‘Benihoppe’) at the white fruit stage with 5–10 cm pedicels were harvested. Uniform fruits were selected, and their pedicels were inserted into 10-mL tubes containing sterile water. Each treatment was injected with 10 fruits. Each fruit was injected with 2 mL of resuspended bacterial solution. The VIGS experiment was repeated independently three times. One week later, Quantitative real-time PCR (qRT-PCR) was used to assess the silencing efficiency in VIGS and Mock fruits. *FaActin* was selected as the reference gene, with three replicates performed, and relative expression levels were calculated using the 2^^−ΔΔCt^ method. Primers are listed in Supplementary Data Set4.

### Determination of anthocyanin and soluble sugar contents

For metabolite analysis, three independent biological replicates were used per treatment, each consisting of pooled tissue from three fruits. Each biological replicate was measured with three technical replicates.

#### Anthocyanin measurement

For metabolite analysis, three strawberry fruits were randomly selected from each treatment group and the corresponding control group, and used as biological replicates. Anthocyanin, glucose, sucrose, and fructose were extracted from individual fruit samples. Mature strawberry fruits were ground with liquid ammonia, and 0.2 g of the fruit powder was taken and mixed with 2 mL of 1% hydrochloric acid methanol. The sample was then placed in a dark environment at 4 °C for 24 h. After that, it was centrifuged at 4 °C and 12,000 rpm for 1 min. 1% hydrochloric acid methanol was used as the control. The absorbance values of the solution at wavelengths of 530 and 657 nm were measured using a spectrophotometer. Anthocyanins were measured using the pH differential method [48]. The calculation formula is as follows: Total anthocyanin content (µg/g) = ((A530 - A657) × D / m) × 1000 D: Dilution factor m: Fruit mass (g).

#### Sugar analysis

Soluble sugars were extracted using ultrapure water as the extraction solvent. The ripe fruit from each treatment group was ground in liquid nitrogen. Weigh 3 g and place it in a 10 mL centrifuge tube containing ultrapure water. After cooling to room temperature, centrifuge at 12,000 rpm for 10 min. Take the supernatant and make it up to 10 mL with deionized water. Take 1 mL of the solution and filter it through a 0.22 μm water system filter membrane. Collect the filtrate for testing. The measurement was performed using an LC-2050 Rid high-performance liquid chromatograph equipped with a 5 μm, 250 mm × 4.6 mm NH2 column. The column temperature was set to 35 °C, and the detector cell temperature was set to 75 °C. An isocratic elution was carried out with a mobile phase of acetonitrile and deionized water (80:20, V/V) at a flow rate of 1 mL/min. A 10 µL aliquot of the sample was injected for analysis, and three replicates were performed [49]. The calculation methods of sucrose, fructose, and glucose are presented in Table 1.

**Table 1:** The calculation methods of sucrose, fructose, and glucose

Component	Regression equation	Correlation coefficient R2
Sucrose	y=55641x + 2215.2	0.992
Fructose	y=554647x + 30,298	0.998
Glucose	y=57315x + 31,904	0.998

### Low-temperature treatment and qRT-PCR

Normally, the plants of cultivar ‘Benihoppe’ at the vegetable stage were subjected to a 4 °C low-temperature treatment under SD (9.5 h/14.5 h day/night) and weak light conditions. Strawberry leaves were sampled before treatment (0 h) and after treatment at 1 h, 3 h, 6 h, 12 h, and 24 h for RNA extraction. qRT-PCR was used to measure the expression levels of *FaCOL* genes at each time point post-treatment, all normalized to the expression level of *FaActin*, Fxa7Ag02220 [50].

### Protein sequence alignment and interaction analysis

The protein sequences of AtCO, AtCOL1, AtCOL2, and AtCOL9, the Arabidopsis proteins with the highest homology to strawberry FaCOL57 and FaCOL59, were obtained via BLAST from The Arabidopsis Information Resource (https://www.arabidopsis.org). Sequence alignment was performed using the website(http://www.novopro.cn/tools). The STRING database (https://cn.string-db.org) was used to predict the protein-protein interaction network for the *FaCOL*s.

### Statistical analyses

All quantitative data were presented as mean ± standard deviation. Statistical analyses were performed using Prism 10. Pairwise comparisons were determined using Student’s *t*-test (*, *P* < 0.05; **, *P* < 0.01; ***, *P* < 0.001).

## Results

### Genome-wide identification and phylogenetic characterization of *FaCOL* genes in cultivated strawberry

**Fig. 1:**
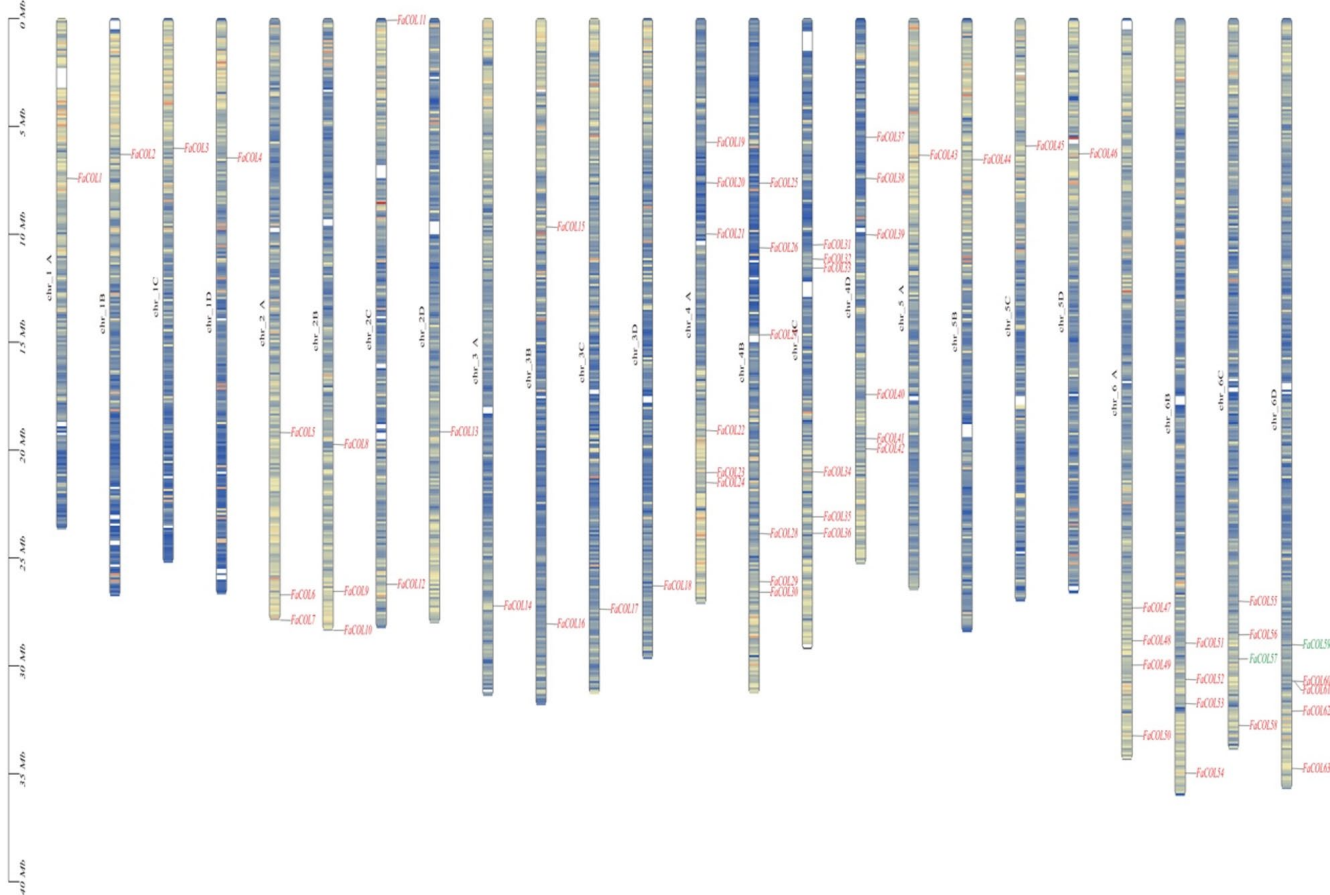
Distributions of 63 *FaCOLs* on 24 chromosomes in cultivated strawberry. Short black vertical lines indicate the position of each *FaCOL*. The deeper the yellow color, the higher the gene density; the deeper the blue color, the lower the gene density

A total of 63 *CONSTANS*-LIKE (*COL*) genes containing conserved B-box and CCT domains were identified in the cultivated strawberry genome (Fig. 1). Detailed gene names and identifiers are provided in Supplementary Data Set 1. Based on their chromosomal positions, these genes were designated *FaCOL1* to *FaCOL63*. The expansion of *FaCOL* members relative to diploid species suggests that gene duplication events associated with polyploidization contributed to COL family diversification in cultivated strawberry. The *FaCOL* genes were distributed across 24 chromosomes with uneven density. Chromosomes 4 A–4D harbored the highest number of *FaCOL* members, each containing six genes (Fig. 1). Nevertheless, at least one *FaCOL* gene was detected on every chromosome. This genome-wide distribution pattern suggests that *COL* genes are broadly retained across the four subgenomes and have likely undergone strong conservation during the polyploidization processes leading to the formation of the octoploid strawberry genome.

Physicochemical characteristics of FaCOL proteins, including coding sequence (CDS) length, amino acid number, molecular weight (MW), isoelectric point (pI), and predicted subcellular localization, are summarized in Supplementary Table S1. Among the identified proteins, FaCOL61 was the shortest, encoding 112 amino acids, whereas FaCOL40 was the longest, consisting of 886 amino acids. Predicted molecular weights ranged from 12.65 kDa (FaCOL61) to 96.14 kDa (FaCOL22), while theoretical pI values varied between 4.45 (FaCOL39) and 9.45 (FaCOL58). Subcellular localization analysis predicted that all FaCOL proteins are nuclear-localized, consistent with their expected roles as transcription factors (Supplementary Data Set 2).

**Fig. 2:**
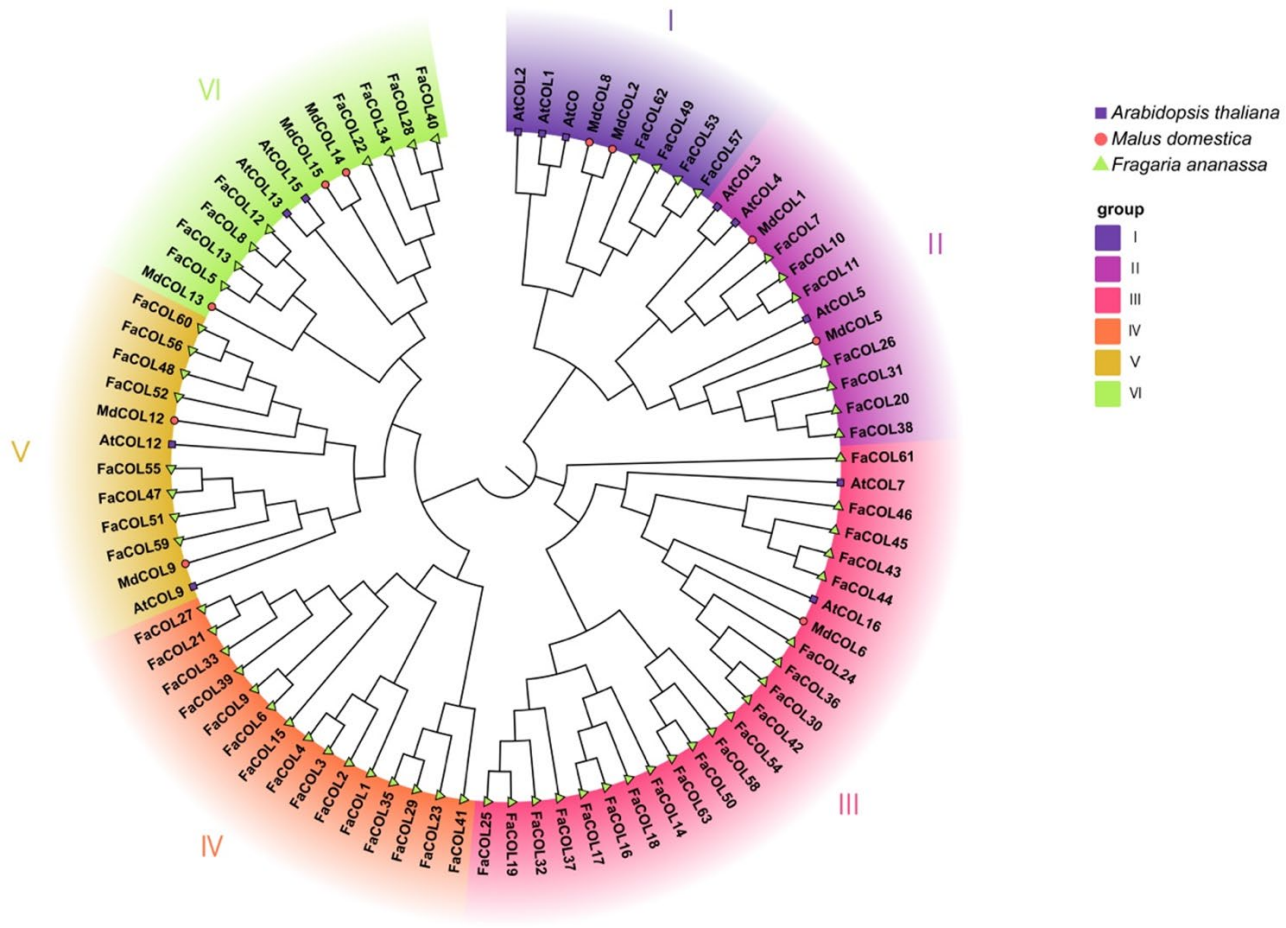
Phylogenetic analysis of FaCOL proteins from *Fragaria* x *ananassa* ‘Benihoppe’, apple, and Arabidopsis. The phylogenetic tree was drawn using NJ method with a bootstrap value of 1000

To investigate evolutionary relationships, a Neighbor-Joining phylogenetic tree was constructed using full-length COL protein sequences from *Arabidopsis thaliana*, apple (*Malus domestica*), and cultivated strawberry. Phylogenetic analysis grouped the FaCOL proteins into three major clades comprising six subtypes (I–VI) (Fig. 2). Members including FaCOL53, FaCOL57, FaCOL49, and FaCOL62 clustered with AtCO, AtCOL1, AtCOL2, MdCOL2, and MdCOL8 within subtype I, indicating potential functional conservation. In contrast, FaCOL59 showed higher sequence similarity with AtCOL9 and MdCOL9, grouping into subtype V (Fig. 2). Compared with Arabidopsis and apple, the FaCOL family exhibited clear lineage-specific expansion in cultivated strawberry, with several proteins forming distinct subclades, particularly within subtype IV (Fig. 2), possibly reflecting genome duplication events associated with strawberry polyploidization.

### Gene structure and conserved motifs of *FaCOLs*

**Fig. 3:**
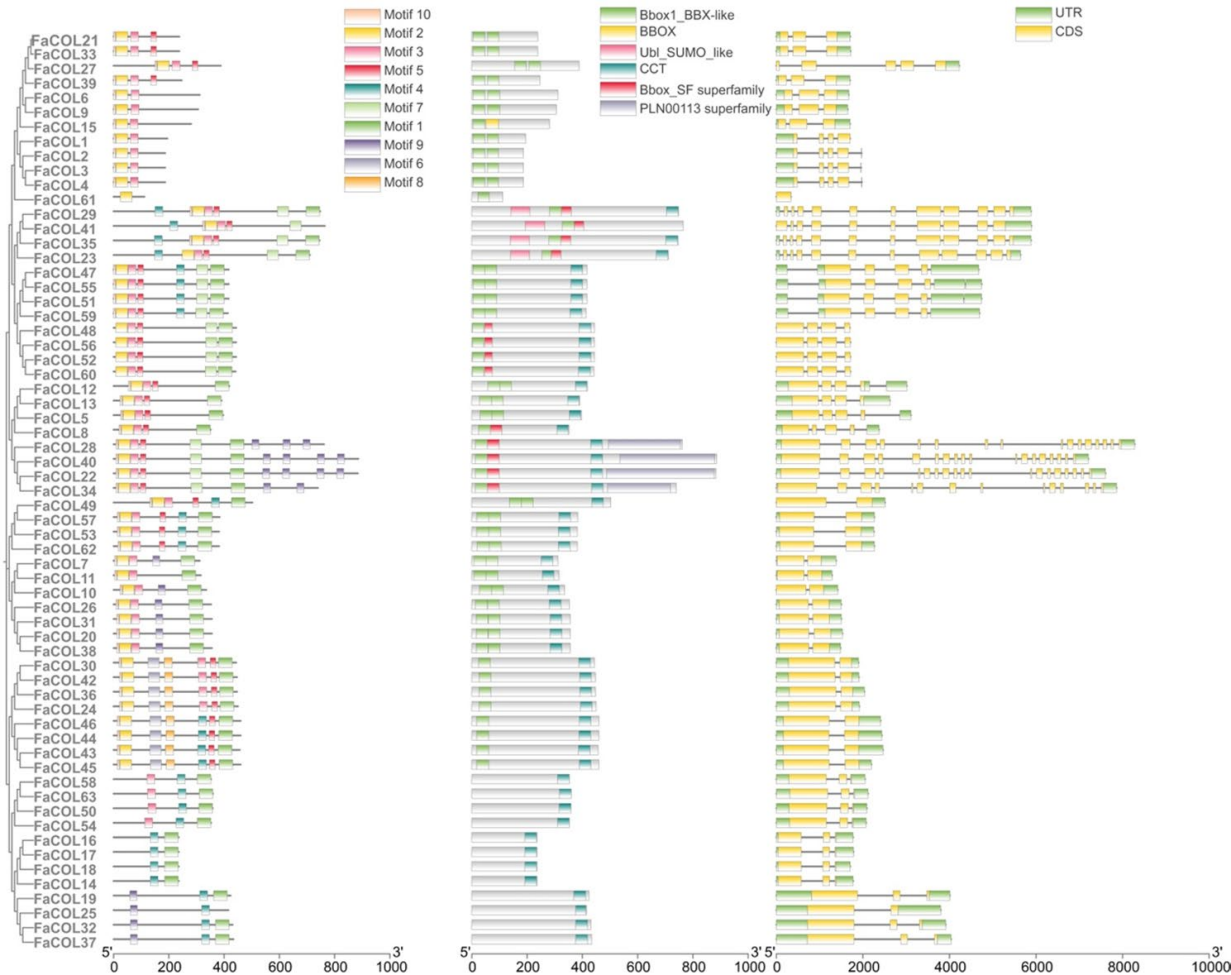
The conserved motifs and gene structure analysis of *FaCOL* genes in cultivated strawberry

To further explore structural diversity within the FaCOL family, exon–intron organization and conserved motif composition were analyzed in the context of phylogenetic relationships. MEME analysis identified ten conserved motifs among FaCOL proteins (Fig. 3; Supplementary Figure S1). Motif 1 and Motif 2 corresponded to the conserved CCT and B-box domains, respectively, and were present in most FaCOL members (Fig. 3 and Supplementary Figure S1). Domain architecture analysis revealed three major structural categories of FaCOL proteins. Type I proteins contained only the CCT domain, Type VI proteins possessed only the B-box domain, whereas Type II proteins contained both domains together with additional Ubl_SUMO-like or B-box_SF superfamily domains (Fig. 1), suggesting increased functional complexity. Gene structure analysis demonstrated that most *FaCOL* genes contained between two and five exons. However, genes harboring Ubl_SUMO-like or B-box_SF domains exhibited more than ten exons (Fig. 3), implying that these additional domains may be associated with alternative splicing or transcriptional regulation diversity. The variation in gene structures and motif compositions suggests potential functional diversification among FaCOL members.

### Cis-element and protein interaction network prediction of *FaCOL*s

**Fig. 4:**
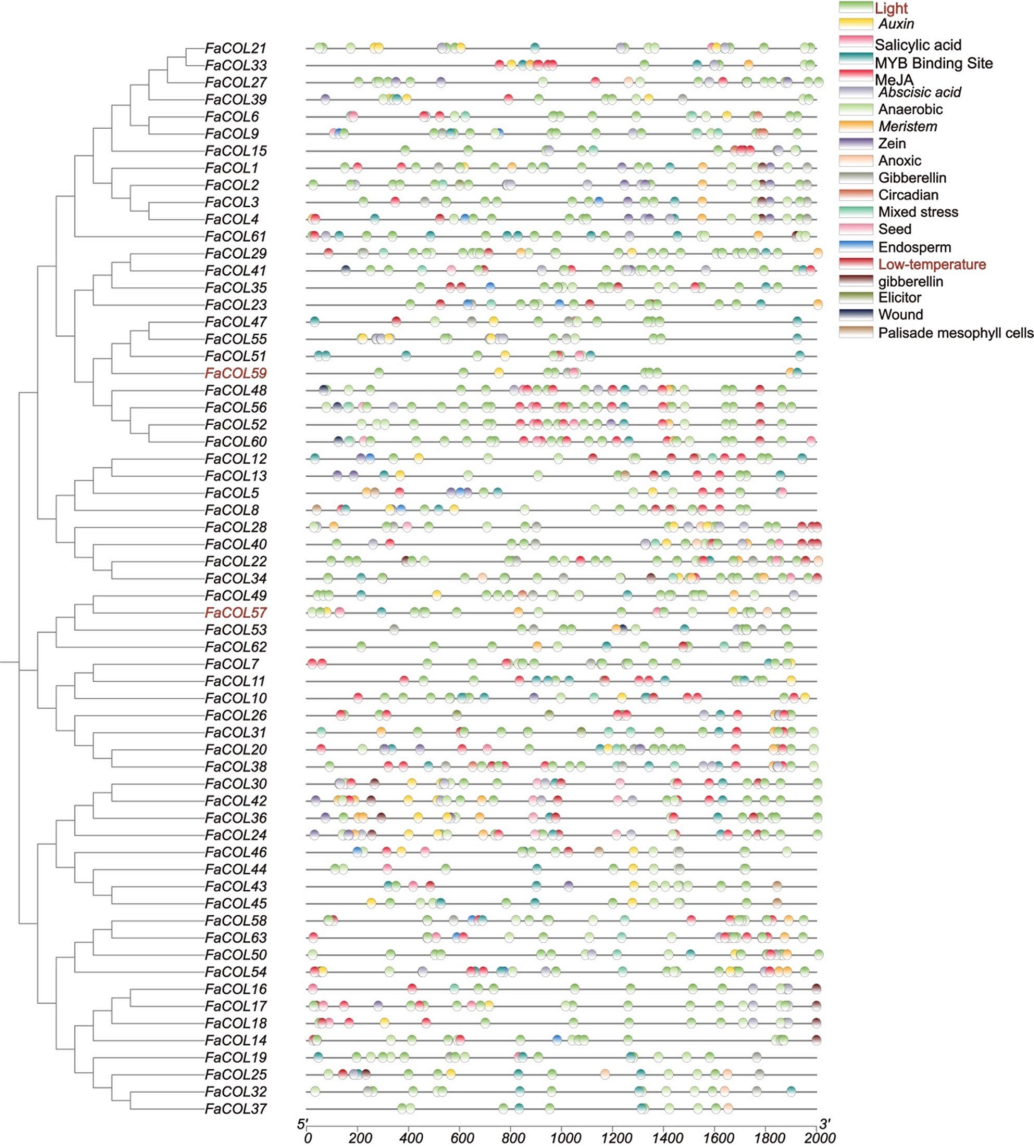
Distribution of the cis-elements in the promoter of *FaCOLs* in cultivated strawberry. Different cis-elements were indicated by different colored symbols

To investigate potential regulatory mechanisms controlling *FaCOL* expression, promoter regions (2 kb upstream of transcription start sites) were extracted and analyzed using PlantCARE. A total of twenty types of cis-regulatory elements were identified (Fig. 4). Light-responsive elements were the most abundant and were present in nearly all *FaCOL* promoters. In addition, numerous elements associated with hormone signaling, including jasmonic acid (JA), abscisic acid, gibberellin, and auxin, as well as abiotic stress-related elements such as drought and low-temperature responsive motifs, were detected. These results suggest that *FaCOL* genes may participate in integrating environmental and hormonal signals.

**Fig. 5:**
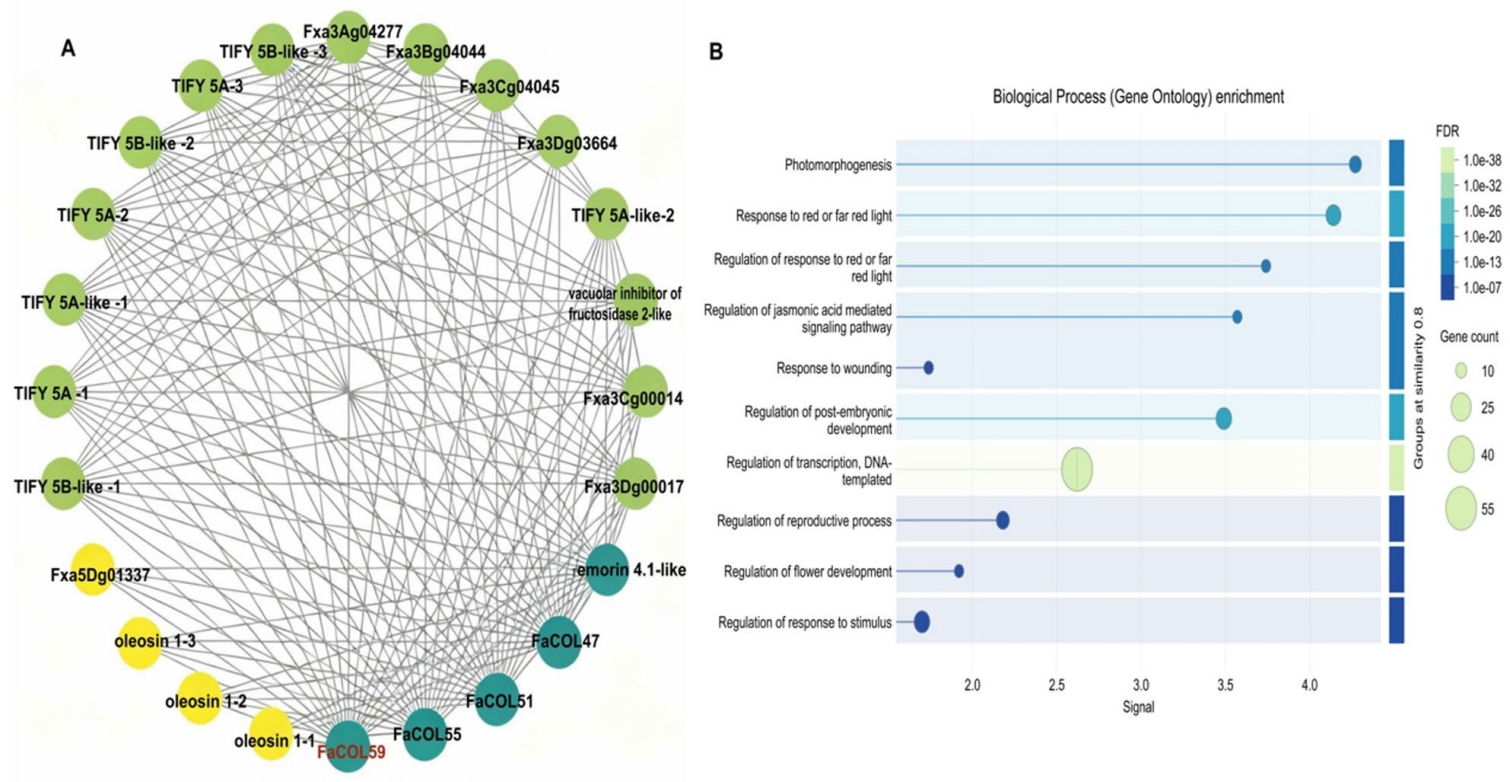
Mapping of the FaCOL protein interaction network. **A**, Prediction of the interaction network associated with FaCOL proteins. **B**, Gene Ontology (GO) enrichment analysis of interacting proteins in (**A**).

To deepen understanding of *FaCOL’s* function, a protein–protein interaction (PPI) network was constructed using the STRING database with a confidence threshold > 0.7; unconnected nodes were excluded. It revealed FaCOL59, FaCOL55, FaCOL51, and FaCOL47 as highly connected to TIFY and oleosin proteins (Fig. 5A). *TIFY* genes regulate growth, hormone signaling, and stress. GO enrichment of interactors implicated *FaCOLs* in transcription, reproduction, flower development, photomorphogenesis, and jasmonic acid signaling (Fig. 5B).

### Collinearity analysis of the COL family

**Fig. 6:**
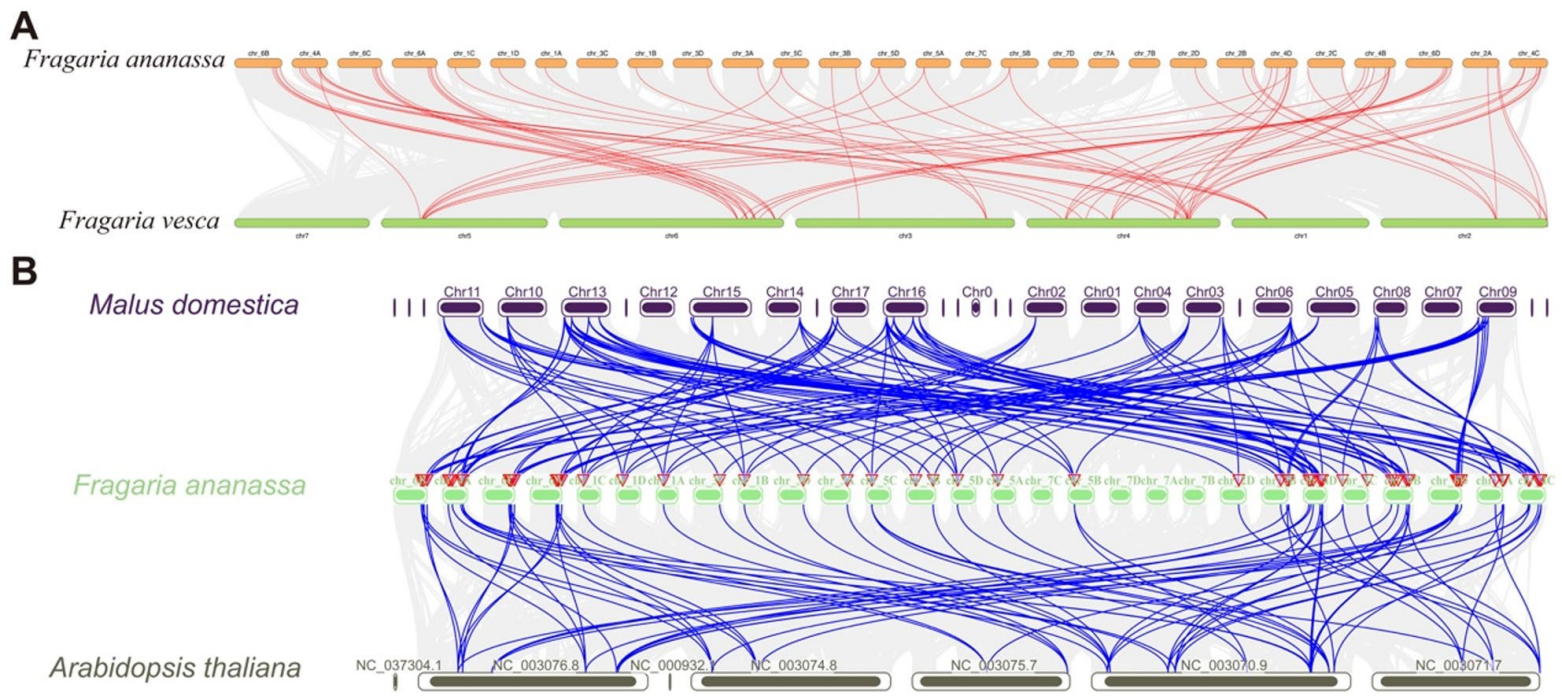
Synteny of COLs among Arabidopsis, apple, and strawberry (**A**). Syntenic relationships of COL genes between the octoploid cultivated strawberry (Fragaria × ananassa) and the diploid woodland strawberry (Fragaria vesca). Red lines denote collinear gene pairs. **B**. Cross-species synteny of COL genes among Arabidopsis thaliana, apple (Malus domestica), and Fragaria × ananassa. Blue lines denote collinear gene pairs.

The cultivated strawberry (*Fragaria* × *ananassa*) is an allo-octoploid species that originated from multiple diploid ancestors through repeated interspecific hybridization and chromosome doubling. Synteny analysis between the octoploid cultivated strawberry and the diploid woodland strawberry (*Fragaria vesca*) identified 68 pairs of *FaCOL* homologous genes. With the exception of chromosome 7 in *F. vesca* and subgenomes 3 C and 7 A–7D in the cultivated strawberry, collinearity of *COL* genes was found in all other chromosomes (Fig. 6A). To investigate the evolutionary history of the *COL* gene family, interspecies synteny among Arabidopsis (5 chromosomes), apple (Malus domestica; 18), and strawberry (28) was identified, yielding 132 strawberry–apple and 78 strawberry–Arabidopsis orthologous pairs (Fig. 6B). This stronger synteny between apple and strawberry reflects their shared Rosaceae ancestry. These results further support the occurrence of an additional lineage-specific whole-genome duplication event in the evolutionary history of the Maleae tribe of Rosaceae (e.g., Malus, Pyrus, and Crataegus).

### Temperature-dependent expression patterns of *FaCOL* genes in strawberry fruits

Given the enrichment of temperature-responsive cis-elements in FaCOL promoters and the temperature sensitivity of strawberry fruit development, transcriptome analysis was conducted to examine *FaCOL* expression under different temperature conditions. Strawberry fruits were incubated under long-day conditions at 6 °C, 16 °C (control), or 26 °C for 6 and 12 days, followed by RNA-seq analysis. Clean reads were mapped to the ‘Benihoppe’ reference genome, and differentially expressed genes (DEGs) were identified using thresholds of |log₂fold change| ≥ 0.585 and FDR < 0.05 (Supplementary Data Set 3).

**Fig. 7:**
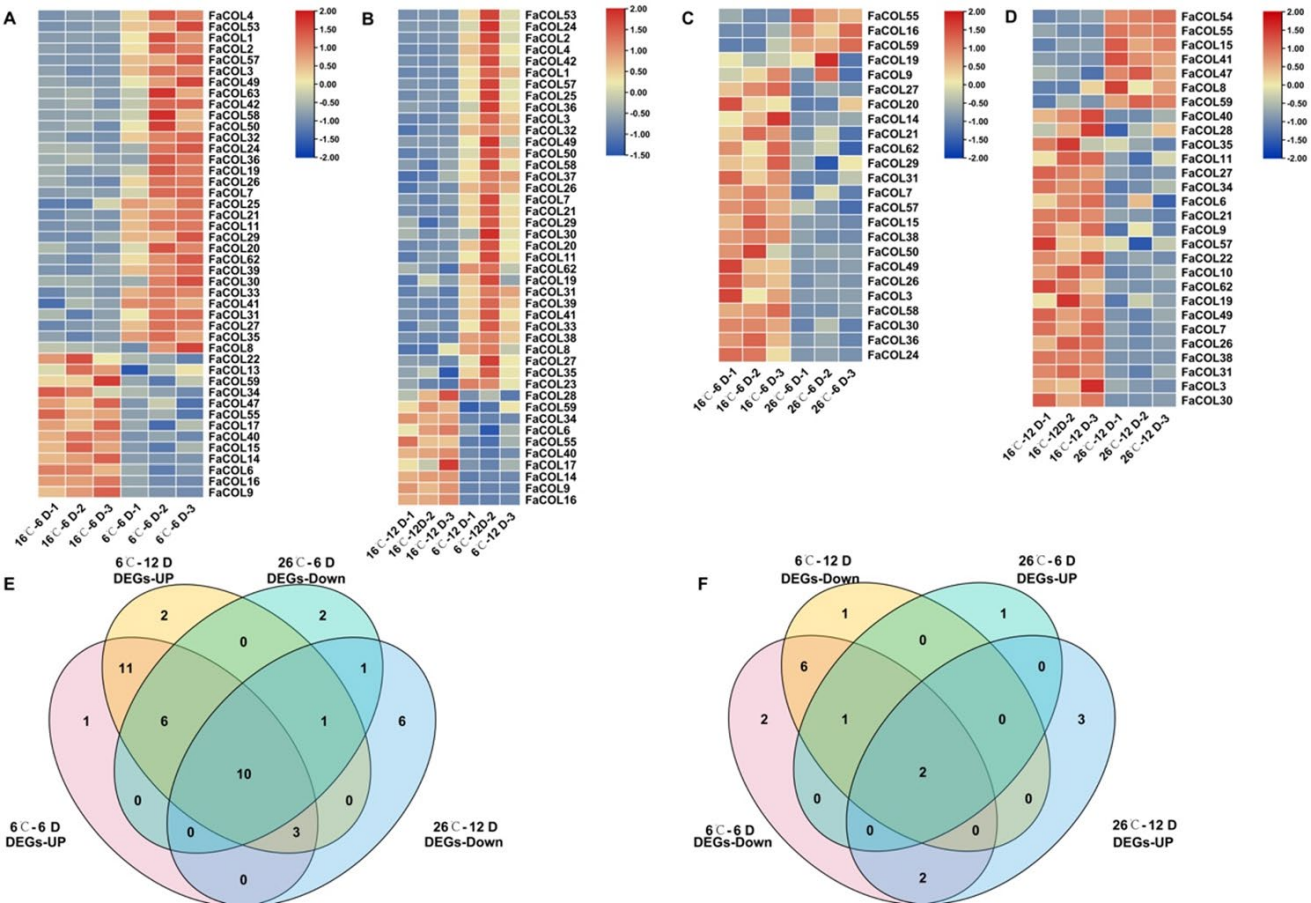
The differentially expressed genes (DEGs) of *FaCOLs* at 26℃ and 6℃ relative to 16℃ in cultivated strawberry fruits. **A**, Heatmap showing expression levels of the DEGs of *FaCOLs* at 6℃, relative to 16℃ for 6 days. **B**, The expression levels of the DEGs of *FaCOLs* at 6℃, relative to 16℃ for 12 days. **C**, Heatmap showing expression levels of the DEGs of *FaCOLs* at 26℃, relative to 16℃ for 6 days. **B**, The expression levels of the DEGs of *FaCOLs* at 26℃, relative to 16℃ for 12 days. **E** Venn diagram showing the number of overlapping genes, which are low-temperature-induced and high-temperature-repressed. **F**, Venn diagram showing the number of overlapping genes, which are low-temperature-repressed and high-temperature-induced. *P* < 0.05

At 6 days, exposure to 26 °C resulted in upregulation of 31 *FaCOL* genes and downregulation of 13 genes (Fig. 7A). Conversely, at 12 days, 26 °C treatment induced four *FaCOL* genes while repressing twenty genes (Fig. 7B). Under cold treatment, 6 °C induced expression of 33 *FaCOL* genes and repressed ten genes after 12 days (Fig. 7C). Meanwhile, 26 °C treatment at 12 days resulted in upregulation of seven genes and downregulation of twenty-one genes (Fig. 7D).

Comparative intersection analysis identified ten *FaCOL* genes (*FaCOL3*, *FaCOL7*, *FaCOL21*, *FaCOL26*, *FaCOL27*, *FaCOL30*, *FaCOL31*, *FaCOL49*, *FaCOL57*, and *FaCOL62*) that were induced by low temperature but repressed under high temperature (Fig. 7E). In contrast, *FaCOL55* and *FaCOL59* exhibited the opposite expression pattern (Fig. 7F). These contrasting expression profiles suggest that specific *FaCOL* members may participate in temperature-dependent regulatory processes during fruit development.

### Opposing temperature-responsive expression patterns of *FaCOL57* and *FaCOL59*

**Fig. 8:**
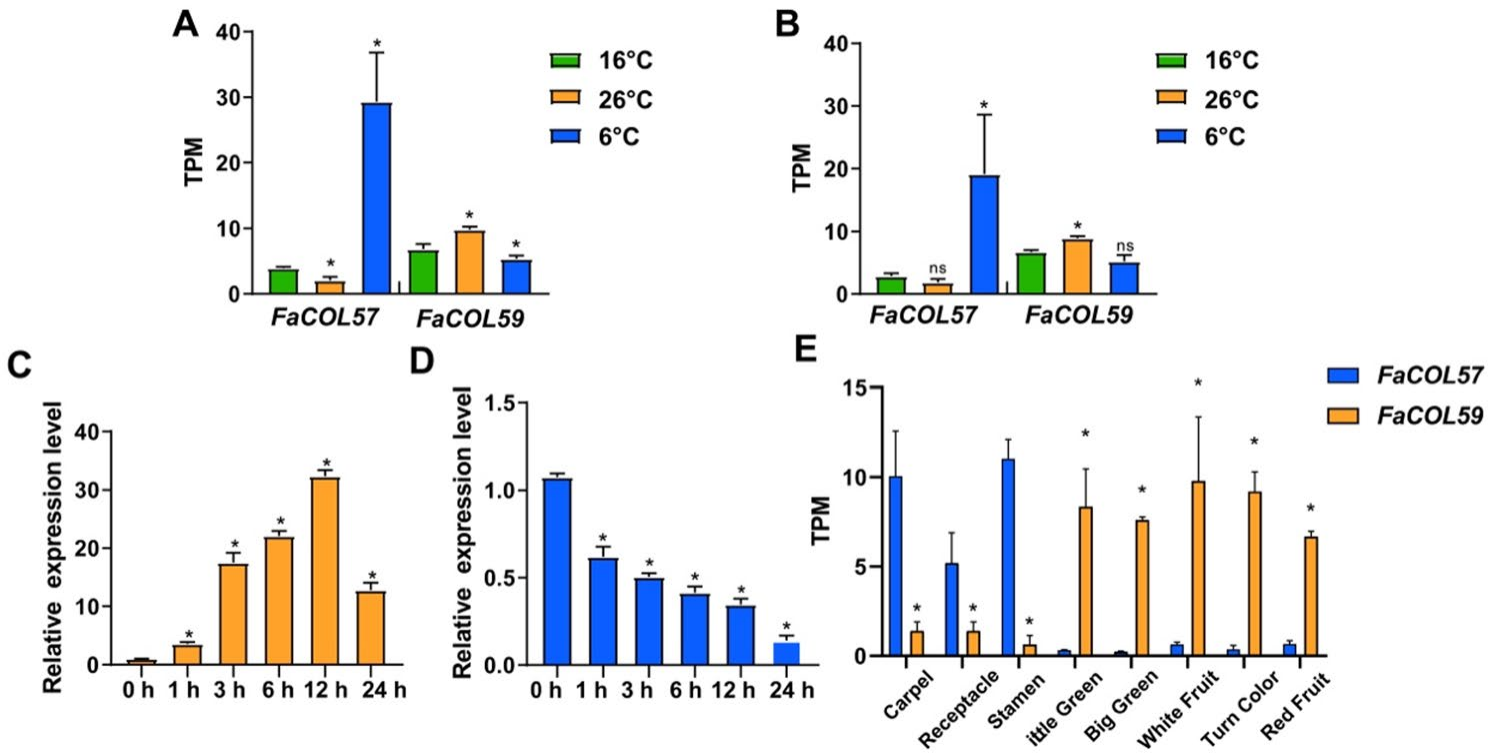
Antagonistic *FaCOL57/FaCOL59* responses patterns in cultivated strawberry. **A**, FaCOL57 and FaCOL59 expression levels at 26℃ and 6℃ (versus 16 °C control) for 6 days. **B**, FaCOL57 and FaCOL59 expression levels at 26℃ and 6℃ (versus 16 °C control) for 12 days. **C**, Relative expression levels of FaCOL57 at 4℃ for different times by qRT-PCR. **D**, Relative expression levels of FaCOL59 in cultivated strawberry fruits at 4℃ for different times by qRT-PCR. **E**, FaCOL57 and FaCOL59 expression levels in different tissues and stages of cultivated strawberry are represented by TPM (Transcripts Per Million) obtained from RNA-seq. Data are mean ± SD from three biological replicates. *, P < 0.05; Student’s t-test

Among temperature-responsive *FaCOL* genes, *FaCOL57* and *FaCOL59* exhibited strikingly opposite expression patterns. Transcriptome data showed that *FaCOL57* expression was significantly suppressed at 26 °C but strongly induced at 6 °C after both 6 and 12 days of treatment (Fig. 8A-B). In contrast, *FaCOL59* displayed the reverse expression pattern, showing induction at 26 °C and repression at 6 °C (Fig. 8A-B). Analysis of publicly available ‘Benihoppe’ transcriptome datasets revealed distinct tissue-specific expression profiles. *FaCOL57* transcripts were highly enriched in floral tissues, including carpels, stamens, and receptacles, but showed relatively low expression in fruit tissues. Conversely, *FaCOL59* exhibited peak expression during fruit ripening stages and relatively low expression in floral organs (Fig. 8E).

To validate cold responsiveness, qRT-PCR analysis was performed using strawberry leaves exposed to 4 °C under short-day conditions. *FaCOL57* expression increased rapidly and reached its peak at 12 h of cold treatment (Fig. 8C), whereas *FaCOL59* expression was significantly suppressed under the same conditions (Fig. 8D). Domain analysis revealed that both *FaCOL57* and *FaCOL59* contain conserved dual B-box and CCT domains and share high sequence similarity with Arabidopsis *CO*/*COL* homologs (Supplementary Figure S2). These results suggest that *FaCOL57* and *FaCOL59* may perform distinct functions in strawberry developmental processes.

### Functional roles of *FaCOL57* and *FaCOL59* in regulating anthocyanin and sugar accumulation

**Fig. 9:**
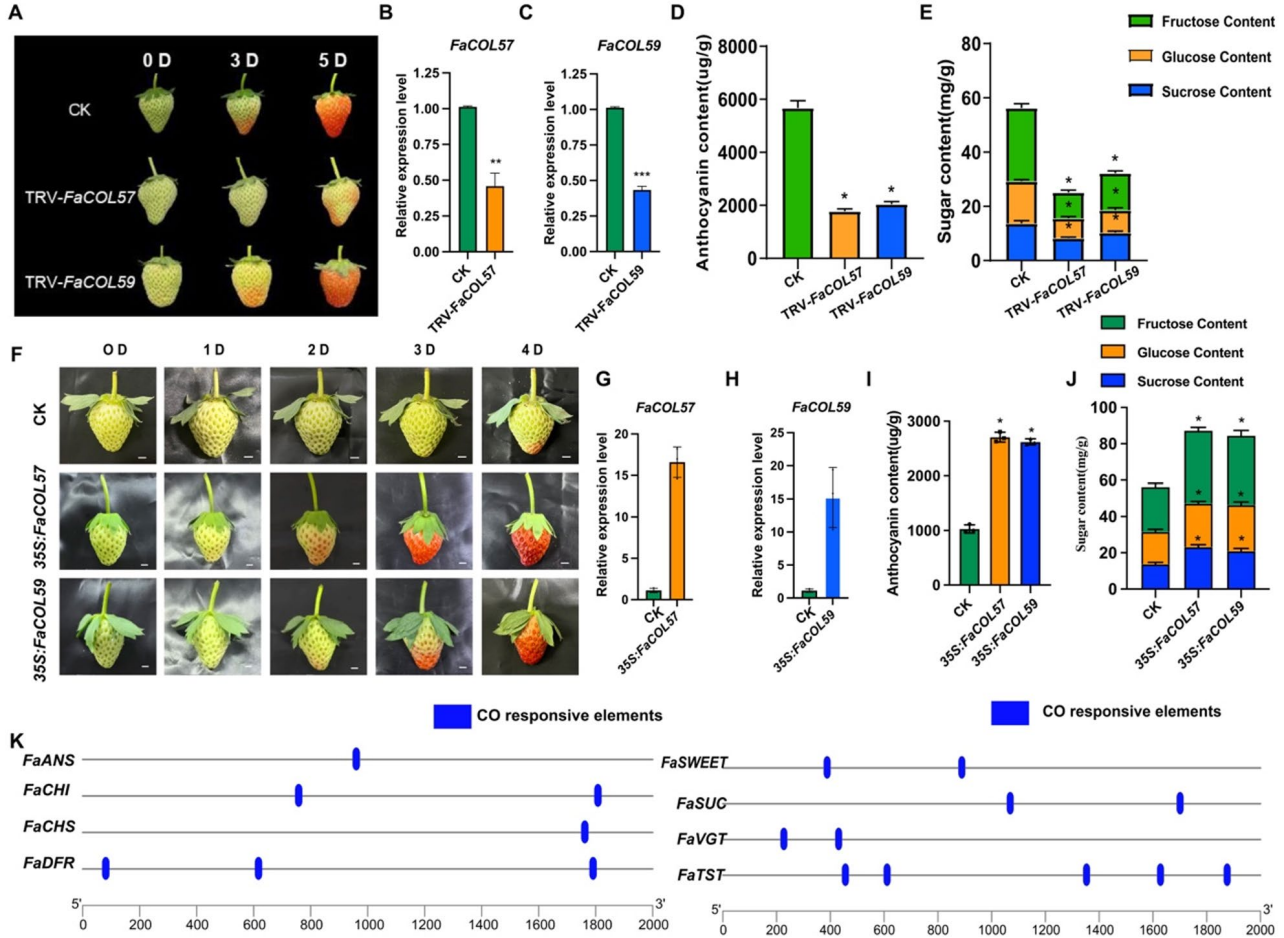
*FaCOL57* and *FaCOL59* positively regulate anthocyanin and soluble sugar accumulation. **A**, Phenotypes of strawberry fruits: Silencing of the FaCOL57 and FaCOL59 genes by VIGS. **B**-**C**, the expression levels of FaCOL57 and FaCOL59 were significantly silenced in the fruit of pTRV-FaCOL57 and pTRV-FaCOL59. **D**, The anthocyanin content in mock, pTRV-FaCOL57, and pTRV-FaCOL59. **E**, The fructose, glucose, and sucrose content in CK, pTRV-FaCOL57, and pTRV-FaCOL59. **F**, A representative fruit for CK and FaCOL57 and FaCOL59 overexpression lines out of 10 analyzed over 4 days. Scale bars, 0.5 cm. **G**-**H**, the expression levels of FaCOL57 and FaCOL59 were significantly enhanced in the fruit of control, 35 S: FaCOL57 and 35 S: FaCOL59 transgenic lines. **I**, The anthocyanin content in mock, 35 S: FaCOL57 and 35 S: FaCOL59. **J**, The fructose, glucose, and sucrose content in CK, 35 S: FaCOL57 and 35 S: FaCOL59. **K**, COL-binding elements of anthocyanin and sugar metabolism genes

To investigate the functional roles of *FaCOL57* and *FaCOL59* in fruit quality formation, virus-induced gene silencing (VIGS) was employed to suppress their expression in strawberry fruits. Fruits infiltrated with *p*TRV-*FaCOL57* or *p*TRV-*FaCOL59* exhibited delayed ripening phenotypes accompanied by reduced anthocyanin accumulation and decreased levels of glucose, fructose, and sucrose compared with control fruits (Fig. 9A–E). To further validate these functional roles, transient overexpression of *FaCOL57* and *FaCOL59* was performed in strawberry fruits. qRT-PCR analysis confirmed significant increases in transcript abundance of both genes. Consistent with VIGS results, overexpression of *FaCOL57* and *FaCOL59* led to significantly elevated levels of anthocyanin and soluble sugars, including sucrose, glucose, and fructose (Fig. 9F–J).

Promoter analysis of key genes involved in anthocyanin biosynthesis (*FaCHI*, *FaCHS*, *FaF3H*, *FaANS*, *FaDFR*) and sugar transport and metabolism (*FaSWEET*, *FaSUG*, *FaTST*) revealed the presence of multiple COL-binding motifs, including CORE, G-box, and GGATTCTC elements (Fig. 9K). These findings suggest that *FaCOL57* and *FaCOL59* may participate in transcriptional regulation of genes involved in fruit pigmentation and sugar metabolism.

## Discussion

### Genome expansion and functional diversification of the *FaCOL* gene family

We identified 63 *FaCOL* genes in octoploid strawberry based on conserved B-box and CCT domains, naming them *FaCOL1*–*FaCOL63* by chromosomal position (Figs. 1 and 2). This exceeds counts in diploid relatives (*Fragaria vesca*; 10) and other species (longan, pear, cannabis, pepper; [40, 41, 44, 51]), reflecting polyploidy-driven expansion that likely underpins functional diversification. Structural analyses revealed considerable diversity in B-box domain composition, exon–intron organization, and conserved motif distribution among FaCOL proteins (Figs. 2 and 3), indicating that structural divergence may contribute to functional specialization. Promoter sequences (2 kb upstream) harbor 20 cis-element classes, predominantly light-responsive, with others tied to circadian rhythms, hormones (auxin, gibberellin, jasmonic acid), and stresses (low temperature, wounding; Fig. 4). These findings suggest that FaCOL transcription factors likely serve as integrative regulatory nodes linking environmental cues to developmental and metabolic pathways. Although COL proteins are traditionally associated with photoperiodic flowering control, our results indicate that *FaCOL* genes may also participate in temperature-responsive regulatory networks in strawberry.

### Temperature-responsive divergence of *FaCOL57* and *FaCOL59*

Transcriptome analysis revealed that *FaCOL57* and *FaCOL59* exhibit antagonistic expression responses under low- and high-temperature treatments. *FaCOL57* was strongly induced under cold conditions but suppressed by elevated temperatures, whereas *FaCOL59* displayed the opposite regulatory pattern (Figs. 7 and 8A and B). Moreover, qRT-PCR under 4 °C stress (0–24 h) confirmed *FaCOL57* upregulation (peak at 12 h) and *FaCOL59* repression (Fig. 8C, D) under cold exposure. Distinct phylogenetic clades and motif compositions likely underlie these patterns. These contrasting expression dynamics suggest functional divergence between paralogous *FaCOL* genes. Previous studies have demonstrated that *COL* genes are involved in light-responsive regulation. Our results further indicate that the strawberry *COL* gene is also responsive to temperature signals.

Phylogenetic analysis showed that *FaCOL57* shares high sequence similarity with *AtCO*, *AtCOL1*, and *AtCOL2* (Fig. 2), which are well-characterized regulators of photoperiodic flowering. Blast against the octoploid genome flags *FaCOL57* as the closest *AtCO* ortholog. *AtCO*, the first identified *COL* gene, integrates light and circadian signals to activate the downstream gene *FT* [16], thereby inducing flowering—a function relatively conserved across species. For example, under LD conditions, *AtCO* overexpression advances flowering in Arabidopsis (LD plant) [52]. In woodland strawberry and rice (SD plant), *FvCO* and *Hd1*, the ortholog of *AtCO*, promote flowering under SD conditions [43]. In grape, *VvCOs* are expressed during floral induction [37], suggesting the function of *CO* gene may be conserved in triggering the floral transition. Moreover, *CONSTANS-LIKE 5* is a key regulator of flower opening and scent emission in *N. attenuata* and *Petunia axillaris* [53]. Expression profiling demonstrated that *FaCOL57* is preferentially expressed in floral organs and reproductive tissues (Fig. 8E), supporting a potential role in regulating flowering-related processes. Previous studies have shown that *CO* orthologs in multiple species regulate floral transition by activating *FT* homologs under inductive environmental conditions. Therefore, *FaCOL57* may represent a conserved component of flowering regulatory pathways that also responds to temperature signals in cultivated strawberry. However, direct functional validation through flowering phenotype analysis remains necessary to confirm this hypothesis.

In contrast, *FaCOL59*, which is closely related to *AtCOL9*, exhibited strong expression during fruit ripening stages (Fig. 8E). *AtCOL9*, *OsCOL9*, and *BnaCOL9* have been reported to modulate flowering time [39, 54–56], suggesting that *FaCOL59* may have evolved specialized regulatory functions associated with fruit development and metabolic regulation. The differential spatial expression patterns of *FaCOL57* and *FaCOL59* further support the possibility that *FaCOL* gene family expansion in octoploid strawberry contributed to functional diversification across reproductive developmental stages.

### *FaCOL57* and *FaCOL59* promote anthocyanin and soluble sugar accumulation in cultivated strawberry

Functional analysis using VIGS demonstrated that suppression of *FaCOL57* or *FaCOL59* significantly reduced anthocyanin accumulation and soluble sugar contents in strawberry fruits (Fig. 9A–E). Conversely, transient overexpression of both genes enhanced anthocyanin biosynthesis and sugar accumulation (Fig. 9F–J), indicating that these transcription factors positively regulate anthocyanin and soluble sugar accumulation. Promoter analysis revealed the presence of canonical COL-binding elements in key structural genes involved in anthocyanin biosynthesis and sugar transport (Fig. 9, E), but direct DNA binding remains to be experimentally validated. These findings suggest that FaCOL transcription factors may may potentially metabolic gene expression. Similar regulatory mechanisms have been reported in other fruit crops. For example, apple and strawberry both belong to Rosaceae. *MdCOL6* binds G-box elements in *MdANS* and *MdUFGT* to drive anthocyanin [22]; Additionally, *PpCOL8* in sand pear interacts with *PpMADS* through the salicylic acid (SA) signaling pathway and plays a crucial role in fruit senescence [57]; the tomato transcription factor SlCOL4 is likely to interact with abscisic acid (ABA) and ethylene signaling pathways and functions as a negative regulator of tomato fruit ripening [34]. In addition to apples, sand pear, and tomato, these observations support the notion that COL transcription factors play conserved roles in coordinating fruit developmental and metabolic processes across species. Moreover, the function of COL proteins, involved in plant morphogenesis and abiotic stress responses, has been reported [32–38]. For instance, *OsCOL16* delays flowering under SD conditions [16, 39]. In soybeans, *COL* genes positively regulate tolerance to salt and drought stress [33], indicating that the *COL* genes have undergone functional diversification across different species.

### Proposed model for *FaCOL*-mediated integration of cold signals and fruit metabolism

**Fig. 10:**
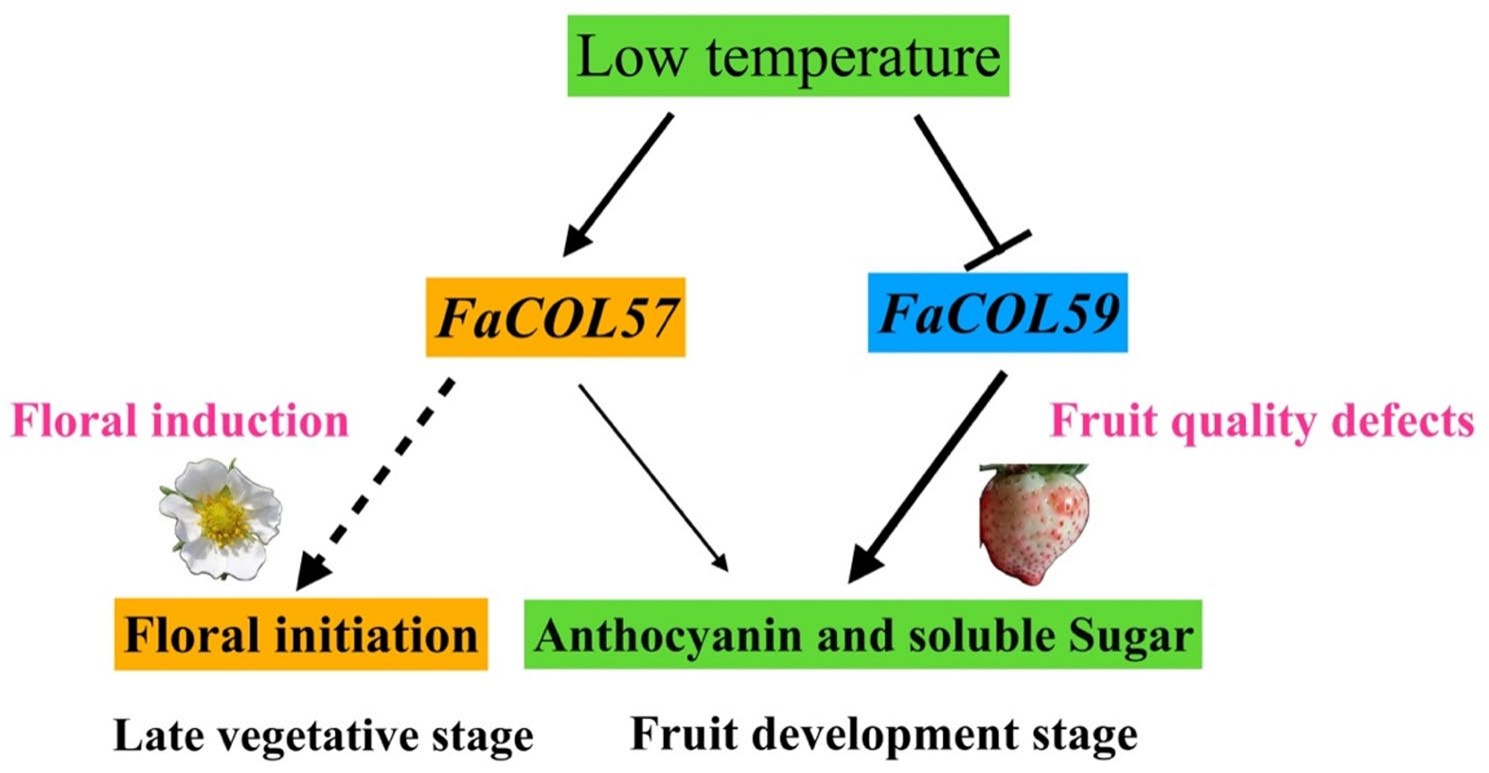
Proposed model illustrating the potential roles of *FaCOL57* and *FaCOL59* in integrating cold signals with fruit quality regulation in cultivated strawberry

By integrating the transcriptomic, environmental responsiveness, and functional validation, we provide a mechanistic foundation for future dissection of temperature-associated reproductive development and fruit quality regulation in strawberry. *FaCOL57* appears to function primarily in reproductive tissue development and may contribute to low-temperature-associated floral transition. In contrast, *FaCOL59* appears to be associated with fruit ripening-related metabolic processes under optimal growth temperatures. Under cold stress, repression of *FaCOL59* may contribute to reduced anthocyanin and sugar accumulation, whereas induction of *FaCOL57* may partially compensate for metabolic regulation (Fig. 10).

Although *FaCOL57* and *FaCOL59* exhibit clear temperature-responsive transcriptional dynamics and positively regulate anthocyanin and soluble sugar accumulation in fruit tissues, it should be noted that the current evidence does not directly demonstrate temperature-dependent functional modulation at the protein level. Our functional assays were conducted under standard growth conditions; therefore, the mechanistic linkage between temperature perception and downstream metabolic regulation remains inferential. We propose that temperature-induced transcriptional reprogramming of *FaCOL* genes may alter their regulatory influence on metabolic pathways, thereby contributing to fruit quality modulation under variable thermal environments. However, direct validation of temperature-dependent promoter binding, protein stability, or transcriptional activity will require chromatin immunoprecipitation assays and stable transgenic lines grown under controlled temperature regimes. Moreover, several questions remain unresolved. Direct functional analyses of *FaCOL57* in low-temperature-induced floral induction are needed to confirm this model by stable transgenic lines. Moreover, whether *FaCOL57* and *FaCOL59* directly bind promoters of anthocyanin and sugar pathway genes requires verification by chromatin immunoprecipitation assays. We are interested in further investigating how *FaCOL57* and *FaCOL59* coordinate low-temperature and photoperiod regulation of flower initiation and fruit quality. Elucidating these regulatory mechanisms will contribute to improving strawberry breeding strategies aimed at enhancing fruit quality and environmental resilience under fluctuating climate conditions.

Cold signals are proposed to differentially regulate *FaCOL57* and *FaCOL59*, which may function as transcriptional nodes linking cold responsiveness to metabolic pathways controlling anthocyanin biosynthesis and soluble sugar accumulation during fruit development. *FaCOL57* is predominantly associated with reproductive tissue development and may contribute to low-temperature–related floral processes, which requires further functional confirmation. Whereas *FaCOL59* appears to function in fruit ripening–associated metabolic regulation under optimal conditions. Repression of *FaCOL59* under cold conditions may contribute to reduced anthocyanin and sugar accumulation, while induction of *FaCOL57* may partially compensate for metabolic regulation. The direct mechanistic links between temperature perception and metabolic regulation remain to be experimentally validated.

## Supplementary Information

Supplementary Material 1.

## Data Availability

The data is contained within the article and Supplementary Materials. The RNA-seq datasets generated during the current study are available in the [https://ngdc.cncb.ac.cn/gsa/s/9rccrS98](https:/ngdc.cncb.ac.cn/gsa/s/9rccrS98) with the accession number GSA: CRA035004.

## Abbreviations

LD: Long days
SD: Short days
qRT-PCR: Quantitative real-time PCR
DEGs: Differentially expressed genes
