# Supplementary figures and images for: Genome-wide identification of CONSTANS-LIKE genes and functional analysis of FaCOL57 and FaCOL59 in regulating anthocyanin and sugar synthesis in cultivated strawberry
Source: BMC Genomics. 2026 Mar 26;27:436. doi: 10.1186/s12864-026-12778-9 (PMC13141299; doi:10.1186/s12864-026-12778-9)

motif1

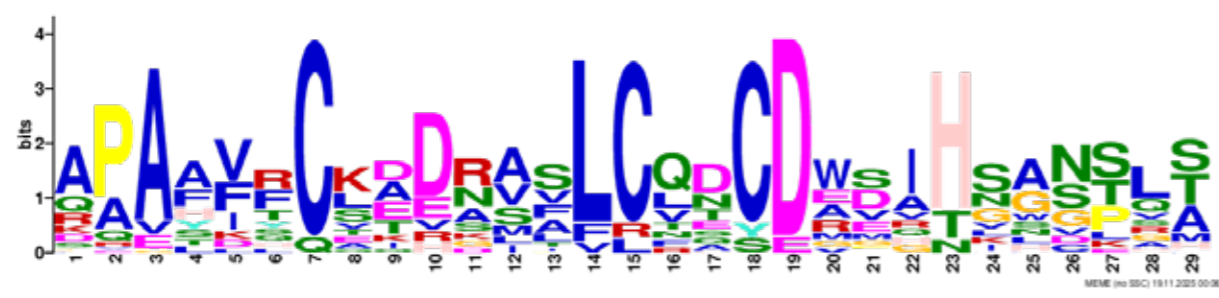

motif6

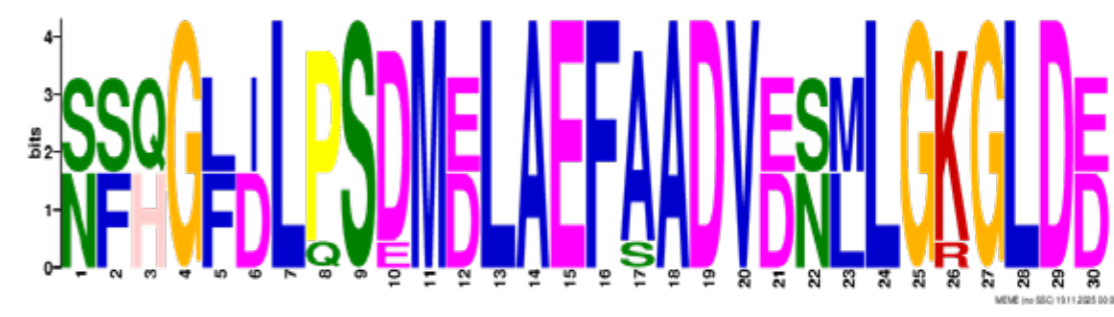

motif2

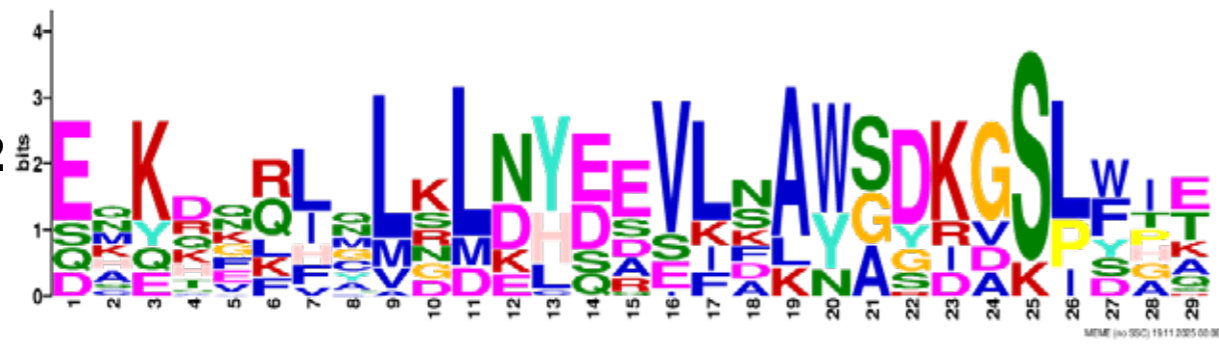

motif7

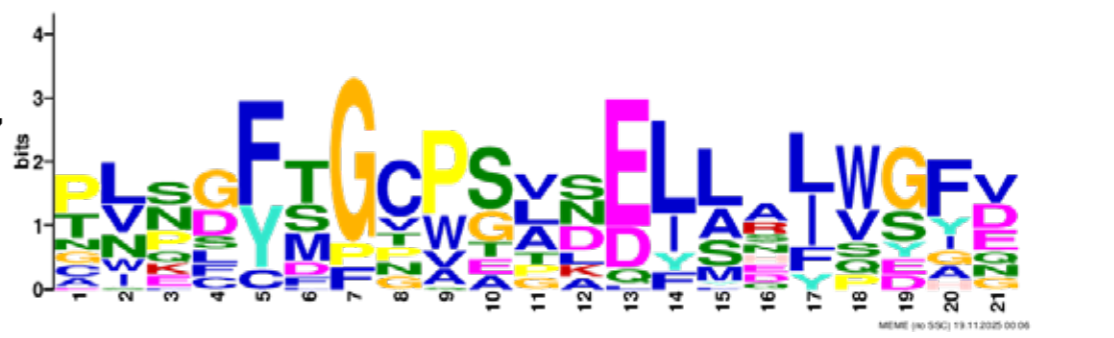

motif3

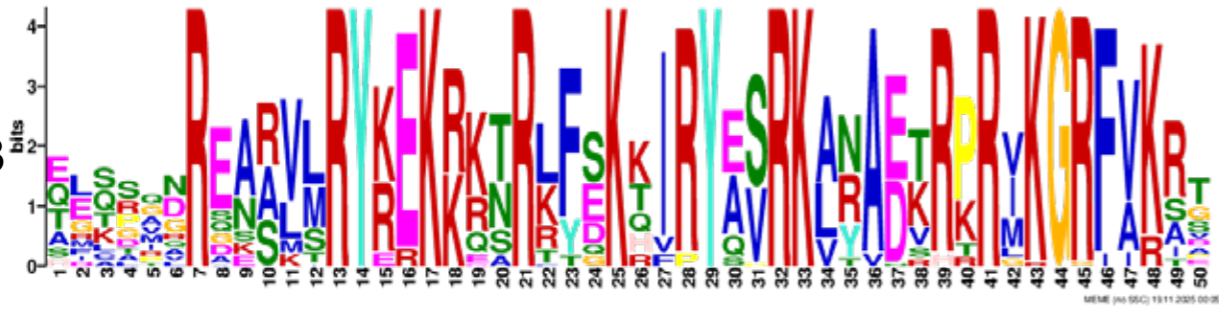

Supplement: Supplementary file 1 — Supplementary Material 1. [file 12864_2026_12778_MOESM1_ESM.zip › Supplementary material-2026/Supplementary Figure S1.pdf]

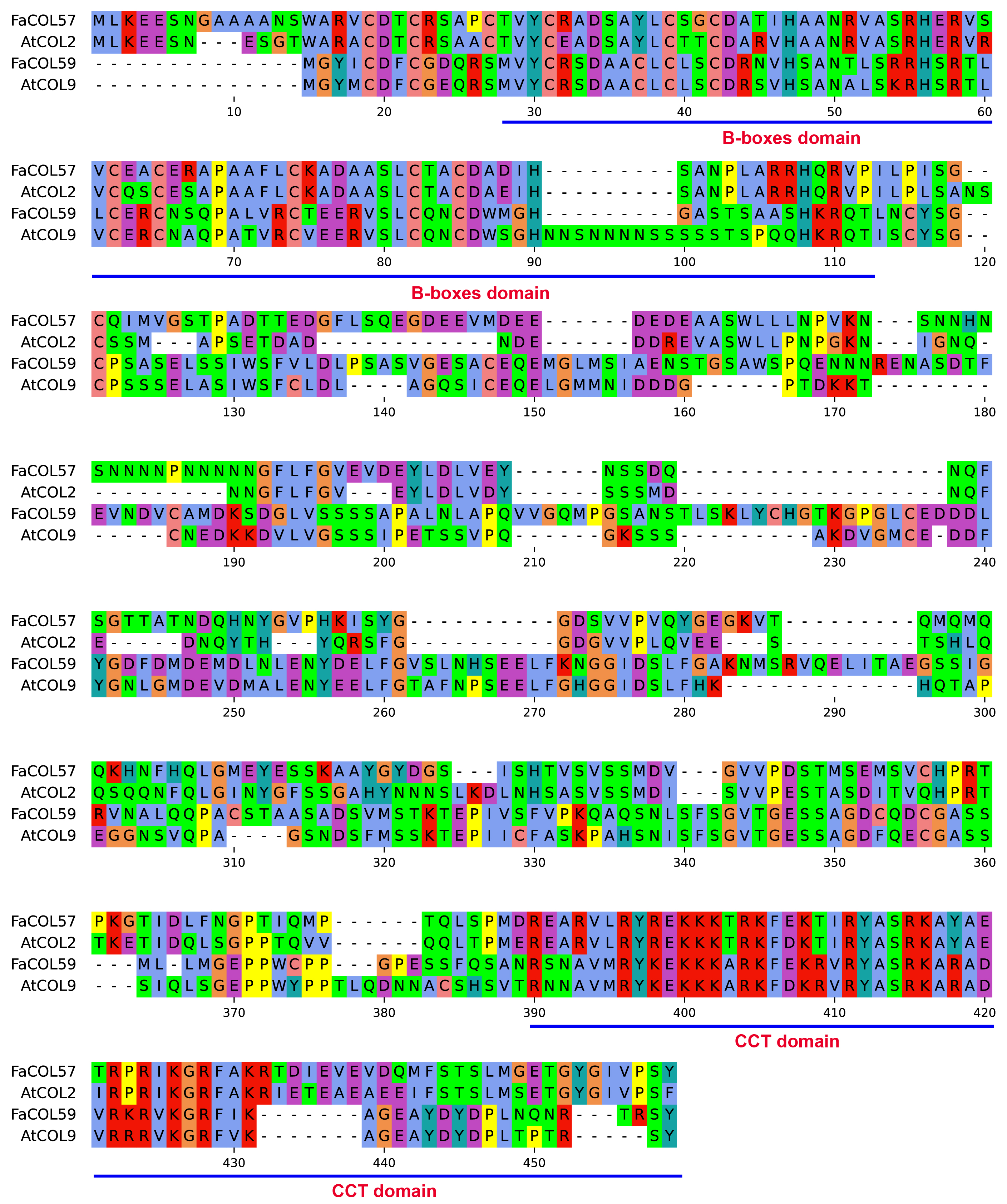

Supplement: Supplementary file 1 — Supplementary Material 1. [file 12864_2026_12778_MOESM1_ESM.zip › Supplementary material-2026/Supplementary Figure S2.jpg]
